# Epidemiological, Microbiological, and Clinical Characteristics of Multi-Resistant *Pseudomonas aeruginosa* Isolates in King Fahad Medical City, Riyadh, Saudi Arabia

**DOI:** 10.3390/tropicalmed8040205

**Published:** 2023-03-30

**Authors:** Taghreed A. Hafiz, Eman A. Bin Essa, Sarah R. Alharbi, Ahmed S. Alyami, Zeina S. Alkudmani, Murad A. Mubaraki, Norah A. Alturki, Fawzia Alotaibi

**Affiliations:** 1 Clinical Laboratory Sciences Department, College of Applied Medical Sciences, King Saud University, Riyadh 12372, Saudi Arabia; 2 Pathology and Clinical Laboratory Medicine, King Fahad Medical City, Riyadh 11525, Saudi Arabia; 3 Pathology Department, College of Medicine, King Saud University, Riyadh 12372, Saudi Arabia

**Keywords:** *Pseudomonas aeruginosa*, respiratory, intensive care unit, nosocomial infection, multi-drug resistant, pan-drug resistant, COVID-19

## Abstract

Increasing rates of serious multi-drug resistant (MDR) *Pseudomonas aeruginosa* infections have been reported globally, including in Saudi Arabia. This retrospective study investigates the epidemiological, microbiological, and clinical characteristics of multi-resistant *P. aeruginosa* (n3579 clinical isolates) in King Fahad Medical City, Riyadh, Saudi Arabia (2019–2021). Information on antimicrobial susceptibility and medical history was collected from the hospital database. *P. aeruginosa* infections occurred in 55.6% of males and 44.4% of females, and *P. aeruginosa* was more prevalent in children than in adults. Our analysis showed that *P. aeruginosa* had the highest sensitivity to amikacin (92.6%) and greatest resistance to aztreonam (29.8%), imipenem (29.5%), ceftazidime (26.1%), meropenem (25.6%), and cefepime (24.3%). MDR and extensively drug resistant (XDR) strains were more prevalent in male than female patients. Female patients showed higher rates of infection with pan-drug resistant (PDR) strains. Respiratory samples contained the majority of resistant isolates. Septic shock and liver disease were strongly correlated with mortality in the ICU patient group after analysing the relative risk associated with mortality. Our study emphasises the threat of multi-resistant *P. aeruginosa* in Saudi Arabia (and potentially the Middle East) and highlights important sources and contexts of infection that inhibit its effective control and clinical management.

## 1. Introduction

*Pseudomonas aeruginosa* is an important Gram-negative opportunistic pathogen found in many environmental settings and can be isolated from different living sources, such as plants, animals, and humans. This organism can persist in both community and hospital settings under conditions of limited nutrition [1]. Community-acquired infections (CAI) are less prevalent than nosocomial infections [2]. The prevalence rate of *P. aeruginosa* isolates in patients with community-acquired pneumonia (CAP) is 3.8% in Europe, 4.3% in North America, 5.2% in Asia, 4.9% in South America, and 5.5% in Africa [3]. *P. aeruginosa* is implicated in various nosocomial infections, including ventilator-associated pneumonia (VAP), bloodstream infections (BSI), urinary tract infections (UTI), and wound infections. It is the fourth most frequently isolated nosocomial pathogen and the second leading contributor to pneumonia, with mortality rates of 27–48% in critically ill patients [4]. Therefore, it is considered as one of the most life-threating bacteria [5].

The emergence of antibiotic resistance in *P. aeruginosa* has become a global public health concern because of the lack of therapeutic alternative treatments available [6], and its ability to metabolize various antibiotics, which promotes infectivity potential [7]. *P. aeruginosa* displays intrinsic and acquired resistance to many antimicrobial agents [8]. According to the European Centre for Disease Prevention and Control’s annual report, 18.7% of all P. aeruginosa isolates were carbapenem resistant and 13.4% were resistant to three or more antimicrobial classes [9]. *P. aeruginosa* can be classified into phenotypes based on drug resistance patterns. Multi-drug resistant (MDR) strains are resistant to three or more anti-pseudomonal drugs, including aminoglycosides, carbapenems, cephalosporins, fluoroquinolones, and penicillin/-lactamase. Extensively drug-resistant (XDR) strains are susceptible to only one or two anti-pseudomonal drugs. Pan-drug resistant (PDR) strains are resistant to all anti-pseudomonal drugs [10].

Infections caused by MDR *P. aeruginosa* strains are associated with high morbidity and mortality rates, as well as increased durations of hospital stays and overall costs of treatment [11,12]. Several factors are associated with MDR *P. aeruginosa* infection, including previous antimicrobial use, prolonged antibiotic treatment, history of chronic obstructive pulmonary disease (COPD), long hospital stay or admission to the intensive care unit (ICU), history of *P. aeruginosa* infection, use of invasive indwelling devices (e.g., tracheostomy tubes or urethral catheters), and previous surgery [13].

A recent increase in the prevalence of MDR and XDR *P. aeruginosa* strains in hospitalised patients has been reported across the globe [6]. In Saudi Arabia, MDR *P. aeruginosa* was most commonly isolated from patients in the ICU [14]. Each year, 13–19% of hospital-acquired infections (HAIs) in the United States are caused by MDR *P*. *aeruginosa* [15]. MDR and XDR isolates are highly prevalent in Europe, particularly in Greece [16]. In a multinational prospective cohort study of 236 ICUs from 77 hospitals in 10 Middle Eastern countries, age, length of stay, central-line days, use of mechanical ventilators, central-line blood stream infection, ventilator associated pneumonia, female gender, and hospitalization were found to be associated with mortality among ICU-admitted patients [17].

The aim of this retrospective study was to examine the epidemiological, microbiological, and clinical characteristics of MDR *P. aeruginosa* isolated at the King Fahad Medical City (KFMC) to inform the development of updated clinical treatment strategies in Saudi Arabia.

## 2. Methods

### 2.1. Study Design

This retrospective study considered data from January 2019 to December 2021 collected at KFMC, Riyadh, Saudi Arabia. A total of 3579 *P. aeruginosa* isolates from various clinical samples were analysed. The clinical history of 255 adult ICU patients was also included in the study.

### 2.2. Data Collection

*P. aeruginosa* isolates were collected from a variety of sources, including blood (central and peripheral lines), the respiratory system (sputum, endotracheal, BAL, nasopharyngeal, throat swab, ear, and eye), urine (mid-stream urine and in-and-out catheters), miscellaneous sources (abscess, wound, tissue, and body fluid), and cerebrospinal fluid (CSF). The inclusion criteria were the same as in the previously published study by Hafiz et al. (2022) [18]. Any bacteria other than *P. aeruginosa* were excluded from this study. ICU patient data (collected from the KFMC database) were included based on their clinical histories as follows: (1) clinical symptoms, such as fever or gastrointestinal tract (GIT) symptoms; (2) wound or urinary tract infections; (3) respiratory diseases, such as COPD, acute respiratory distress syndrome (ARDS), and pulmonary oedema; (4) pneumonia, including ventilator-associated pneumonia (VAP), CAP, and hospital-acquired pneumonia (HAP); (5) mechanical ventilation used at some point; (6) chronic diseases, such as renal disease, heart disease, liver disease, brain disorders, malignancy, diabetes mellitus (DM), asthma, or hypertension (HTN); (6) septicaemia; (7) severe acute respiratory syndrome coronavirus 2 (SARS-CoV-2) infection; (8) clinical outcomes for the patient, and additional notes, if present.

### 2.3. P. aeruginosa Identification and Antimicrobial Susceptibility Testing

For complete identification and sensitivity testing, isolates were confirmed to be *Pseudomonas* spp. using a BD Phoenix automated microbiological system (Becton Dickinson Diagnostic Systems, Sparks, MD, USA). Only data of patients with definitively identified *P. aeruginosa* isolates were included in this study. The following antibiotics were tested for antimicrobial sensitivity testing (AST): ceftazidime, cefepime, ciprofloxacin, piperacillin-tazobactam, gentamicin, amikacin, imipenem, meropenem, colistin, levofloxacin, and aztreonam. The E test was used to confirm the presence of resistant isolates. The results were interpreted and reported in accordance with the CLSI-M100, 32nd edition, and classified as susceptible (S), intermediate (I), or resistant (R). According to the International Consensus, *P. aeruginosa* isolates were classified based on their antibiotic resistance [19].

### 2.4. Statistical Analysis

All data were analysed using SPSS software (IBM SPSS, Armonk, NY, USA). Graphs were generated using GraphPad Prism software (v9.4.1; GraphPad Software, San Diego CA, USA). We compared clinical characteristics among adult ICU-admitted patients over one year using a univariate chi-squared test, with significance set at *p* < 0.05. Similarly, a univariate analysis was performed to compare the outcomes and risk factors associated with mortality among ICU patients. Relative risk (RR) was calculated to determine how the risk of death increased among patients with respiratory infections, and the results were reported as RR and 95% confidence intervals. Antimicrobial susceptibility test results were presented as percentages.

### 2.5. Ethical Consideration

The study protocol was approved by the Ethical Research Committee of King Fahad Medical City. Consent was acquired from the KFMC in accordance with the ICH GCP guidelines (IRB log number: 21-063E).

## 3. Results

### 3.1. Demographic Characteristics of Patients with Multi-Resistant P. aeruginosa

**Figure 1:**
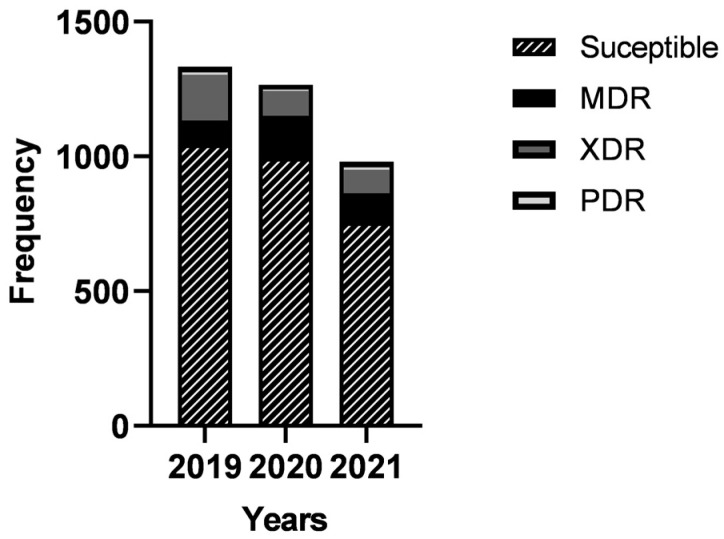
Distribution of multi-resistant *P. aeruginosa* isolates for the period 2019–2021.

Out of the 3579 *P. aeruginosa* isolates, 422 (11.8%) were XDR, 348 (9.7%) were MDR, and 11 (0.3%) were PDR. MDR isolates increased after 2019, whereas the percentages of XDR and PDR isolates decreased (Figure 1).

Most MDR and XDR *P. aeruginosa* isolates (61.8 and 61.1%, respectively) were isolated from male patients, while female patients were infected with more PDR isolates (Table 1). The majority of the MDR isolates (38.8%, 26/67) among paediatrics occurred in infants < 1 year of age, whereas the highest number of XDR isolates (36.8%, 25/68) occurred in paediatric children. PDR isolates were only found in infants, with 100% (2/2) of the paediatric population. Among adult patients, those aged 65–84 years had the highest incidence of MDR (39.5%, 111/281) and XDR isolates (34.5%, 89/258). Additionally, 44.4% (4/11) of PDR isolates occurred in adults aged 45–64 years, 33.3% (3/11) in adults aged 65–84 years, and only two isolates in adults aged 19–44 years. No PDR isolates were observed in adults aged 85 years and above.

The most common sources of MDR and XDR isolates were from respiratory samples (58.9 and 54%, respectively), followed by miscellaneous sources (Table 1). In contrast, a relatively small number of isolates were obtained from urine, blood, and CSF samples. Regarding PDR *P. aeruginosa*, 54.4% (6/11) were isolated from respiratory sources, 27.3% (3/11) from blood, and 18.2% (2/11) from miscellaneous sources. No PDR isolates were obtained from urine and CSF samples.

### 3.2. Antimicrobial Susceptibility of Multi-Resistant P. aeruginosa Isolates

Different isolates showed varying rates of resistance to the respective antibiotics (Table 2). Among MDR and XDR *P. aeruginosa* isolates, the highest rates of resistance were observed against cephalosporins (cefepime and ceftazidime), carbapenems (imipenem and meropenem), and aztreonam. On the other hand, these isolates showed lower rates of resistance to amikacin and colistin. XDR *P. aeruginosa* isolates were highly resistant to ciprofloxacin (90.8%) and levofloxacin (91.2%). PDR *P. aeruginosa* isolates showed 100% resistance to all antibiotics tested.

### 3.3. Clinical Characteristics of ICU Patients Infected with Multi-Resistant P. aeruginosa

During the study period, 255 multi-resistant *P. aeruginosa* isolates were identified from adult patients admitted to the ICU (Table 3). The percentage of ICU patients infected with multi-resistant *P. aeruginosa* was higher for males (61.6%) than that for females (38.4%). The majority of the infected ICU patients (44.3%) were aged 65–84 years. The highest number of multi-resistant *P. aeruginosa* isolates were from respiratory sources (69.8%). Several clinical variables differed between years and were significantly higher in 2019 than in 2020 or 2021, including the incidences of cancer (*p* = 0.006) and liver disease (*p* = 0.001). Conversely, the incidences of pneumonia (*p* < 0.001), mechanical ventilation (*p* < 0.001), and COVID-19 (*p* < 0.001) were significantly higher in 2021 than in 2019 or 2020. Regarding clinical outcomes, mortality rates among infected adults in ICU were high overall (55.7%, 142/255), though mortality rates were highest in 2019. A relatively small number (31/255) of patients were transferred to other hospitals and 32.2% (82/255) were treated.

### 3.4. Clinical Outcomes and Factors Associated with Mortality Rates of ICU Patients Infected with Multi-Resistant P. aeruginosa

Mortality rates were higher among males than females (61.1% and 38.9%, respectively; Table 4). Mortality rates were highest in patients aged 65–84 years (38.9%), followed by those aged 45–64 years (25.7%). The occurrence of septic shock (*p* = 0.001), kidney disease (*p* = 0.038), liver disease (*p* = 0.006), cancer (*p* = 0.035), and mechanical ventilation (*p* = 0.016) was significantly higher in dead patients than that in live patients, while fever (*p* = 0.028) and brain disorders (*p* = 0.002) were significantly higher in live patients. A considerable proportion of ICU patients had a respiratory infection (51.1%). Septic shock (RR = 2.458, CI 95% = 1.080–5.593, *p* = 0.032) and liver disease (RR = 3.107, CI 95% = 1.053–9.162, *p* = 0.039) were the factors most significantly associated with mortality (Table 5).

## 4. Discussion

*P. aeruginosa* is commonly associated with HAIs. The emergence and spread of MDR/XDR *P. aeruginosa* strains are a global public health concern. The prevalence of MDR *P. aeruginosa* has increased globally, comprising 30% of reported *P. aeruginosa* infections in some countries [20]. In Saudi Arabia, *P. aeruginosa* is an increasingly frequent nosocomial pathogen, accounting for 11% of all hospital-acquired infections [21]. In this study, we analysed the epidemiological, microbiological, and clinical characteristics of *P. aeruginosa* isolates. Respiratory samples were the most common source of MDR (58.9%) and XDR (54%) isolates, consistent with previous observations [14,21,22].

XDR *P. aeruginosa* was the most prevalent, followed by MDR and PDR strains. The overall prevalence rate of MDR isolates during the three years was 9.7%, which was higher than the previously reported rate of [23]. This increase coincides with the COVID-19 pandemic (2020–2021), which is consistent with the trends in the prevalence of other MDR bacteria [24,25,26]. Tiri et al., showed that the incidence of carbapenem-resistant *Enterobacterales* colonisation increased from 6.7% in 2019 to 50% in 2020 [24]. Similarly, a study from Jizan, Saudi Arabia, reported a significant increase in the prevalence of carbapenem-resistant enterobacterales (22.4%) during the pandemic period compared to the pre-pandemic period (5.4%) [25]. Additionally, the latest national report from the Centers for Disease Control and Prevention (CDC) reported that, from 2019 to 2020, there was a 35% increase in hospital-onset MDR *P. aeruginosa* infections [26]. This increased incidence could be attributed to the increased selection of resistant strains, owing to the high consumption of antibiotics to treat secondary bacterial infections associated with SARS-CoV-2 infection.

We found that the highest number of *P. aeruginosa* isolates, including MDR and XDR isolates, was collected from patients aged between 65 and 84 years, consistent with the findings of previous studies [6,22]. Older adults are generally at a higher risk of developing nosocomial infections, especially MDR infections, owing to their diminished immune response and increased prevalence of comorbidities, such as cardiovascular disease and diabetes [27]. Additionally, we found that MDR and XDR *P. aeruginosa isolates* were more predominant in males (61%) than females (38.2%). This is consistent with the findings of both national and international studies, showing that the prevalence and drug resistance of Gram-negative pathogens, including *P. aeruginosa*, are higher in males than females [14,22,28,29]. Males are generally more susceptible to bacterial infections due to physiological factors related to sex chromosomes and hormones. The variation between males and females can also be linked to behavioural differences, such as adherence to treatment or smoking rates [28]. Interestingly, we found that PDR isolates were more prevalent in females (63.6%) than males (36.4%), though the causative factors are unclear.

Globally, the overuse of antibiotics has changed the antimicrobial resistance profile of *P. aeruginosa,* resulting in an increase in the prevalence of carbapenemase- or β-lactamase-producing strains and the development of resistance to colistin. We assessed the resistance profiles of *P. aeruginosa* isolates against commonly used antibiotics and observed high resistance rates against monobactams (aztreonam [29.8%]), carbapenems (imipenem [29.5%] and meropenem [25.6%]), and cephalosporins (cefepime [24.3%] and ceftazidime [26.1%]). Overall, XDR *P. aeruginosa* isolates showed higher resistance rates to carbapenems and cephalosporins than the MDR isolates. We found that amikacin and colistin were the most effective antibiotics, with high susceptibility rates of 92.6% and 90.4%, respectively, which is consistent with the findings of previous studies [30,31,32,33,34].

In Saudi Arabia, the overall level of cephalosporin resistance in *P. aeruginosa* is low compared with resistance rates in neighbouring countries (96% and 86% in Qatar and Bahrain, respectively) [23]. A recent nationally-representative survey of *P. aeruginosa* clinical isolates collected in March 2018 and April 2019 reported resistance rates of 14.17% and 8.53% to ceftazidime and cefepime, respectively, with the highest resistance levels observed in Riyadh (19.14% and 10.49%, respectively) [35]. In our study, the resistance rates against cephalosporin were higher than those previously reported [23,35]. We found that the overall resistance rates among all *P. aeruginosa* isolates were 26.1% and 24.4% against ceftazidime and cefepime, respectively. For XDR and MDR isolates, the resistance rates against ceftazidime were 96% and 83%, respectively, whereas the resistance rates against cefepime were 93% and 76%, respectively. This increased resistance rate may be associated with selective pressure exerted by the overuse of broad-spectrum antibiotics, including cephalosporins, to treat or prevent secondary infections in COVID-19 patients [36].

Our study reported high resistance rates against carbapenems, including imipenem and meropenem, of up to 96% among MDR and XDR isolates. This result is of particular concern as carbapenems are the “last resort” drugs to treat severe nosocomial infections caused by MDR Gram-negative bacteria, including *P. aeruginosa* [37]. In Saudi Arabia, the prevalence of infections caused by carbapenemase-producing organisms, mainly Enterobacterales, *Acinetobacter* spp., and *Pseudomonas* spp., has increased in recent years [38,39]. Most of these infections occur in hospitalised patients and are associated with high carbapenem resistance rates. It has been shown that *P. aeruginosa* isolates from UTI samples have a concerning resistance rate of 50% against meropenem and 52.4% against imipenem [23,39]

The emergence and spread of carbapenem resistance has led to the reuse of colistin (a last-resort drug) to treat carbapenem-resistant Gram-negative bacteria, including *P. aeruginosa* [6,40]. We observed a comparatively lower resistance rate against colistin (1.1%). Colistin remains highly effective in the Middle East and North Africa Region, reaching 100% in most countries where some MDR *P. aeruginosa* isolates remain susceptible to colistin [30,41]. The prevalence of colistin resistance has recently increased in Saudi Arabia, reaching 8% and 30% [14,42]. Although low, the level of colistin resistance reported in this study should serve as a warning against the mismanagement of this drug in clinical practice. Therefore, continued surveillance of drug resistance is essential to control the spread of colistin-resistant pathogens.

In this study, 255 multi-resistant *P. aeruginosa* isolates were obtained from adult ICU patients, mainly from respiratory sources (69.8%). Most ICU patients (44.3%) were aged 65-84 years. ICU patients are at high risk of HAIs [43]. In the European Union countries, *P. aeruginosa* is the most common culprit of ICU-acquired pneumonia, UTIs, and bloodstream infections [44]. A comprehensive surveillance study performed in 14 countries of the Middle East demonstrated that, after *Escherichia coli* (7%) and *Klebsiella pneumoniae* (8%), *P. aeruginosa* (5%) was the most common pathogen responsible for peripheral venous catheters-related bloodstream infections (PVCR-BSIs) in ICU patients [45]. In Saudi Arabia, the prevalence of MDR *P. aeruginosa* in ICU patients has remained high over the past few decades (30.6% in 2004–2009 and 35.7% in 2016) [30]. Moreover, a prospective cohort study found that *P. aeruginosa* was responsible for 21.7% of VAP among adult patients in ICU [46]. Several studies reported a high prevalence of antimicrobial-resistant bacterial infections among COVID-19 patients admitted to ICU [24,47]. A recent study from Makkah, Saudi Arabia, showed that a large percentage of bacteria (57.1–76.2%) that infected ICU COVID-19 patients were resistant to multiple antibiotics, including Ampicillin, Ciprofloxacin, Levofloxacin, Imipenem, and Moxifloxacin [48]. We reported an increase in the prevalence of MDR *P. aeruginosa* among ICU COVID-19 patients from 17.9% in 2020 to 36.5% in 2021. Infections with MDR *P. aeruginosa* are often associated with severe adverse clinical outcomes, including an increase in the duration of hospital stay, as well as mortality and morbidity rates [6,49]. In our study, several co-morbidities, including UTI, pneumonia, and septic shock, were identified in ICU patients infected with MDR *P. aeruginosa*, with the most common comorbidities being respiratory tract infections (76%) and pneumonia (45%). Our analysis also showed that mechanical ventilation was strongly associated with MDR *P. aeruginosa* infections in ICU patients during 2020/2021. Mechanical ventilators are widely used in ICUs and are a well-known source of nosocomial infections, mainly VAP [50]. Balkhy et al., showed that, in an adult ICU in Riyadh, VAP-associated *P. aeruginosa* had 13–31% resistance rates to carbapenems, third-generation cephalosporins, fluoroquinolones, aminoglycosides, and antipseudomonal penicillins [51]. Mechanical ventilators are also essential for the respiratory support of critically ill patients with COVID-19, which may explain the increased rates of pneumonia cases in our study during 2020/2021.

The high mortality rates of ICU patients infected with MDR *P. aeruginosa* are a major concern. Mortality rates associated with MDR *P aeruginosa* can be up to four times higher than those associated with non-MDR *P aeruginosa* [52]. Our results demonstrated an overall high mortality rate among ICU patients during the three years (55.7%, 142/255). We observed a decline in the mortality rate from 62.3% in 2019 to 44.9% and 57.1% in 2020 and 2021, respectively. Overall, mortality rates in our study were significantly higher than those reported in USA and Europe (35.7%) [20]. As expected, mortality rates in ICU patients aged 65–84 years old were the highest (38.9%). Furthermore, the mortality rate was significantly higher in patients with septic shock, kidney disease, liver disease, cancer, and those on mechanical ventilation. An alarmingly high percentage of ICU patients had a respiratory infection (51.1%). With further analysis of the relative risk associated with mortality in the ICU patient group, septic shock and liver disease were significantly associated with mortality. The liver plays an essential role in clearing pathogenic bacteria, and there is a high association between liver dysfunction and sepsis. Pre-existing liver dysfunction is a risk factor for developing sepsis and septic shock and is strongly associated with mortality in patients with sepsis. Additionally, sepsis induces liver dysfunction, which is considered a late manifestation of sepsis-induced multiple organ dysfunction syndrome [53]. Our result suggests that patients with pre-existing liver disease and sepsis are at particularly high risk of death due to infection with MDR *P. aeruginosa*.

Our study design has several strengths. The sampling effort comprised a range of diverse sample sources from various sites that enabled an analysis of the type of infection associated with different sources. Additionally, the large sample size of ICU patients allowed us to make confident conclusions. However, the study’s main limitation is that it only considered data from a single tertiary institution. A large-scale multi-centre surveillance system in Riyadh and other cities in Saudi Arabia is needed to fully elucidate the threat of *P. aeruginosa*, among other drug-resistant pathogens.

## 5. Conclusions

Although *P. aeruginosa* can still be treated with anti-pseudomonal drugs, trends in its resistance profile require a large-scale monitoring effort in Saudi Arabia. Our study and the data from recent studies reported an increase in antimicrobial resistance during the COVID-19 pandemic. The observed increase in resistance could be attributed to the extensive and inappropriate use of antibiotics as preventive and therapeutic management of COVID-19, which can increase the selective pressure for antimicrobial resistance [47]. We did not address in our study the antibiotic consumption during the pandemic, nevertheless, we believe that the local pattern of antibiotic prescription during the pandemic is similar to the global pattern, which showed an increase in antibiotic use during the pandemic [54,55]. Our study provides well-supported evidence for MDR *P. aeruginosa* being a high-risk pathogen in Saudi Arabia, especially against older adults and ICU patients.

## Figures and Tables

**Table 1:** Distribution of multi-resistant *P. aeruginosa* isolates according to gender, age, and sample source and site.

Characteristic	Total *n* (%) (Total = 3579)	*P. aeruginosa* Isolates				*p*-Value
		*P. aeruginosa* *n* (%) (Total = 2798)	MDR *n* (%) (Total = 348)	XDR *n* (%) (Total = 422)	PDR *n* (%) (Total = 11)	
*Gender*						
Male	1990 (55.6)	1513 (54.1)	215 (61.8)	258 (61.1)	4 (36.4)	0.002 **
Female	1589 (44.4)	1285 (45.9)	133 (38.2)	164 (38.2)	7 (63.6)	
*Age*						
Paediatric infant (<1 year)	236 (26.2)	185 (24.2)	26 (38.8)	23 (33.8)	2 (100)	0.001 **
Paediatric child (1–10 years)	466 (51.7)	417 (54.6)	24 (35.8)	25 (36.8)	0 (0)	
Paediatric adolescent (11–18 years)	199 (22.1)	162 (21.2)	17 (25.4)	20 (29.4)	0 (0)	
Adult (19–44 years)	658 (24.6)	491 (24.1)	59 (21)	83 (32.2)	2 (22.2)	0.022 *
Adult (45–64 years)	889 (33.2)	695 (34.2)	83 (29.5)	80 (31)	4 (44.4)	
Adult (65–84 years)	968 (36.1)	728 (35.8)	111 (39.5)	89 (34.5)	3 (33.3)	
Adult (≥85 years)	163 (6.1)	120 (5.9)	28 (10)	6 (2.3)	0 (0)	
*Source*						
Miscellaneous	927 (25.9)	738 (26.4)	79 (22.7)	108 (25.6)	2 (18.2)	<0.001 **
Blood	284 (7.9)	232 (8.3)	24 (6.9)	25 (5.9)	3 (27.3)	
Respiratory	1633 (45.6)	1194 (42.7)	205 (58.9)	228 (54)	6 (54.5)	
Urine	719 (20.1)	622 (22.2)	39 (11.2)	58 (13.7)	0 (0)	
CSF	16 (0.4)	12 (0.4)	1 (0.3)	3 (0.7)	0 (0)	

* Statistically significant; the number of * represents the degree of significance. CSF, cerebrospinal fluid; MDR, multi-drug resistant; XDR, extensively drug-resistant; PDR, pan-drug resistant.

**Table 2:** Comparison of antimicrobial susceptibility among multi-resistant *P. aeruginosa* isolates.

Antibiotics	Total *n* (%) (Total = 3579)	*P. aeruginosa* Isolates				*p*-Value
		*P. aeruginosa* *n* (%) (Total = 2798)	MDR *n* (%) (Total = 348)	XDR *n* (%) (Total = 422)	PDR *n* (%) (Total = 11)	
*Ceftazidime*						
Sensitive	2529 (70.7)	2498 (89.3)	25 (7.2)	6 (1.4)	0 (0)	<0.001 **
Intermediate	117 (3.3)	78 (2.8)	31 (8.9)	8 (1.9)	0 (0)	
Resistant	933 (26.1)	222 (7.9)	292 (83.9)	408 (96.7)	11 (100)	
*Cefepime*						
Sensitive	2617 (73.1)	2567 (91.7)	42 (12.1)	8 (1.9)	0 (0)	<0.001 **
Intermediate	92 (2.6)	33 (1.2)	39 (11.2)	20 (4.7)	0 (0)	
Resistant	870 (24.3)	198 (7.1)	267 (76.7)	394 (93.4)	11 (100)	
*Ciprofloxacin*						
Sensitive	2783 (77.8)	2554 (91.3)	218 (62.6)	11 (2.6)	0 (0)	<0.001 **
Intermediate	159 (4.4)	95 (3.4)	36 (10.3)	28 (6.6)	0 (0)	
Resistant	637 (17.8)	149 (5.3)	94 (27)	383 (90.8)	11 (100)	
*Piperacillin-Tazobactam*						
Sensitive	2573 (71.9)	2516 (89.9)	44 (12.6)	13 (3.1)	0 (0)	<0.001 **
Intermediate	394 (11)	178 (6.4)	114 (32.8)	102 (24.2)	0 (0)	
Resistant	612 (17.1)	104 (3.7)	190 (54.6)	307 (72.7)	11 (100)	
*Gentamicin*						
Sensitive	3085 (86.2)	2709 (96.8)	274 (78.7)	102 (24.2)	0 (0)	<0.001 **
Intermediate	77 (2.2)	38 (1.4)	12 (3.4)	27 (6.4)	0 (0)	
Resistant	417 (11.7)	51 (1.8)	62 (17.8)	293 (69.4)	11 (100)	
*Amikacin*						
Sensitive	3313 (92.6)	2776 (99.2)	317 (91.1)	220 (52.1)	0 (0)	<0.001 **
Intermediate	66 (1.8)	7 (0.3)	9 (2.6)	50 (11.8)	0 (0)	
Resistant	200 (5.6)	15 (0.5)	22 (6.3)	152 (36)	11 (100)	
*Imipenem*						
Sensitive	2365 (66.1)	2270 (81.1)	85 (24.4)	10 (2.4)	0 (0)	<0.001 **
Intermediate	158 (4.4)	139 (5)	16 (4.6)	3 (0.7)	0 (0)	
Resistant	1056 (29.5)	389 (13.9)	247 (71)	409 (96.9)	11 (100)	
*Meropenem*						
Sensitive	2555 (71.4)	2446 (87.4)	99 (28.4)	10 (2.4)	0 (0)	<0.001 **
Intermediate	108 (3)	88 (3.1)	15 (4.3)	5 (1.2)	0 (0)	
Resistant	916 (25.6)	264 (9.4)	234 (67.2)	407 (96.4)	11 (100)	
*Colistin*						
Sensitive	3234 (90.4)	2546 (91)	308 (88.5)	380 (90)	0 (0)	<0.001 **
Intermediate	305 (8.5)	241 (8.6)	33 (9.5)	31 (7.3)	0 (0)	
Resistant	40 (1.1)	11 (0.4)	7 (2)	11 (2.6)	11 (100)	
*Levofloxacin*						
Sensitive	2587 (72.3)	2412 (86.2)	155 (44.5)	20 (4.7)	0 (0)	<0.001 **
Intermediate	261 (7.3)	179 (6.2)	65 (18.7)	17 (4)	0 (0)	
Resistant	729 (20.4)	207 (6.4)	126 (36.2)	385 (91.2)	11 (100)	
*Aztreonam*						
Sensitive	2124 (59.3)	2043 (73)	54 (15.5)	27 (6.4)	0 (0)	<0.001 **
Intermediate	381 (10.6)	306 (10.9)	40 (11.5)	35 (8.3)	0 (0)	
Resistant	1065 (29.8)	442 (15.8)	253 (72.7)	359 (85.1)	11 (100)	

* Statistically significant; the number of * represents the degree of significance.

**Table 3:** Characteristics of ICU patients with multi-resistant *P. aeruginosa* infection for the period 2019–2021.

Characteristic	Total *n* (%) (Total = 255)	Years			*p*-Value
		2019 *n* (%) (Total = 114)	2020 *n* (%) (Total = 78)	2021 *n* (%) (Total = 63)	
*Gender*					
Male	157 (61.6)	66 (57.9)	52 (66.7)	39 (61.9)	0.470
Female	98 (38.4)	48 (42.1)	26 (33.3)	24 (38.1)	
*Age*					
Adult (19–44 years)	54 (21.2)	33 (28.9)	11 (14.1)	10 (15.9)	0.256
Adult (45–64 years)	72 (28.2)	29 (25.4)	24 (30.8)	19 (30.2)	
Adult (65–84 years)	113 (44.3)	46 (40.4)	38 (48.7)	29 (46)	
Adult (⇒85 years)	16 (6.3)	6 (5.3)	5 (6.4)	5 (7.9)	
*Source of specimen*					
Miscellaneous	31 (12.2)	12 (10.5)	14 (17.9)	5 (7.9)	0.003 **
Blood	26 (10.2)	12 (10.5)	3 (3.8)	11 (17.5)	
Respiratory	178 (69.8)	75 (65.8)	56 (71.8)	47 (74.6)	
Urine	20 (7.8)	15 (13.2)	5 (6.4)	0 (0)	
*Clinical presentation/infection*					
Fever	34 (13.4)	18 (15.8)	12 (15.4)	4 (6.3)	0.182
Sepsis	25 (9.8)	9 (7.9)	7 (9)	9 (14.3)	0.375
Septic shock	40 (15.7)	22 (19.3)	5 (6.4)	13 (20.6)	0.025 *
Respiratory	195 (76.5)	102 (89.5)	63 (80.8)	30 (47.6)	<0.001 **
GIT	8 (3.1)	5 (4.4)	2 (2.6)	1 (1.6)	0.558
UTI	32 (12.5)	16 (14)	12 (15.4)	4 (6.3)	0.222
WI	46 (18)	24 (21.1)	12 (15.4)	10 (15.9)	0.529
Pneumonia	117 (45.9)	27 (23.7)	38 (48.7)	52 (82.5)	<0.001 **
*Underlying disease*					
Kidney disease	90 (35.3)	42 (36.8)	28 (35.9)	20 (31.7)	0.787
Heart disease	101 (39.6)	44 (38.6)	35 (44.9)	22 (34.9)	0.465
Liver disease	32 (12.5)	26 (22.8)	4 (5.1)	2 (3.2)	<0.001 **
Brain disorder	68 (26.7)	34 (29.8)	24 (30.8)	10 (15.9)	0.082
Cancer	33 (12.9)	23 (20.2)	4 (5.1)	6 (9.5)	0.006 **
DM	136 (53.3)	56 (49.1)	44 (56.4)	36 (57.1)	0.478
Asthma	8 (3.1)	4 (3.5)	2 (2.6)	2 (3.2)	0.934
HTN	152 (59.6)	55 (48.2)	54 (69.2)	43 (68.3)	0.004 **
*Risk factors*					
Mechanical ventilation	93 (36.5)	23 (20.2)	24 (30.8)	46 (73)	<0.001 **
COVID-19	37 (14.5)	0 (0)	14 (17.9)	23 (36.5)	<0.001 **
*Outcome*					
Died	142 (55.7)	71 (62.3)	35 (44.9)	36 (57.1)	0.002 **
Treated	82 (32.2)	35 (30.7)	24 (30.8)	23 (36.5)	
Transferred	31 (12.2)	8 (7)	19 (24.4)	4 (6.3)	

* Statistically significant; the number of * represents the degree of significance. GIT, gastrointestinal tract; UTI, urinary tract infection; WI, wound infection; DM, diabetes mellitus; HTN, hypertension; COVID-19, coronavirus disease 2019.

**Table 4:** Comparison of demographic and clinical characteristics among dead and alive ICU patients infected with multi-resistant *P. aeruginosa*.

Characteristic	Outcome		*p*-Value
	Alive *n* (%) (Total = 113)	Dead *n* (%) (Total = 142)	
*Year*			
2019	43 (38.1)	71 (50)	0.056
2020	43 (38.1)	35 (24.6)	
2021	27 (23.9)	36 (25.4)	
*Gender*			
Male	69 (61.1)	88 (62)	0.882
Female	44 (38.9)	54 (38)	
*Age*			
Adult (19–44 years)	32 (28.3)	22 (15.5)	0.075
Adult (45–64 years)	29 (25.7)	43 (30.3)	
Adult (65–84 years)	44 (38.9)	69 (48.6)	
Adult (≥85 years)	8 (7.1)	8 (5.6)	
*Respiratory culture*			
Positive	87 (48.9)	91 (51.1)	0.026 *
*Clinical presentation/infection*			
Fever	21 (18.6)	13 (9.2)	0.028 *
Sepsis	9 (8)	16 (11.3)	0.378
Septic shock	8 (7.1)	32 (22.5)	0.001 **
Respiratory	80 (70.8)	115 (81)	0.057
GIT	5 (4.4)	3 (2.1)	0.293
UTI	15 (13.3)	17 (12)	0.755
WI	23 (20.4)	23 (16.2)	0.391
Pneumonia	56 (49.6)	61 (43)	0.293
*Underlying disease*			
Kidney	32 (28.3)	58 (40.8)	0.038 *
Heart	46 (40.7)	55 (38.7)	0.749
Liver	7 (6.2)	25 (17.6)	0.006 **
Brain disorder	41 (36.3)	27 (19)	0.002 **
Cancer	9 (8)	24 (16.9)	0.035 *
DM	53 (46.9)	83 (58.5)	0.066
Asthma	6 (5.3)	2 (1.4)	0.076
HTN	70 (61.9)	82 (57.7)	0.497
*Risk factors*			
Mechanical ventilation	23 (28.3)	61 (43)	0.016 *
COVID-19	15 (3.3)	22 (15.5)	0.617

Univariate analysis (Fisher’s exact test, *p* < 0.05). * Statistically significant; the number of * represents the degree of significance. GIT, gastrointestinal tract; UTI, urinary tract infection; WI, wound infection; DM, diabetes mellitus; HTN, hypertension; COVID-19, corona virus disease 2019.

**Table 5:** Relative risk associated with mortality among adult ICU patients infected with multi-resistant *P. aeruginosa* isolates.

Variable	RR	CI 95%	*p*-Value
Septic shock	2.458	1.080–5.593	0.032 *
Kidney disease	1.287	0.851–1.946	0.232
Liver disease	3.107	1.053–9.162	0.039 *
Cancer	1.673	0.738–3.789	0.217
Mechanical ventilation	1.219	0.828–1.796	0.314

* Statistically significant; CI, confidence interval; RR, relative risk. RR < 1 indicates no association with mortality, RR >1 indicates a strong association with mortality.

## Data Availability

Data available in the KFMC Institute data system and could be available for public upon special request.
